# Supplementary material for: Chromothripsis during telomere crisis is independent of NHEJ, and consistent with a replicative origin
Source: Genome Res. 2019 May;29(5):737–49. doi: 10.1101/gr.240705.118 (PMC6499312; doi:10.1101/gr.240705.118)
Supplement: Supplemental Material [file supp_gr.240705.118_Supplemental_file_1.zip › contigs/annotated_contigs/DB79/contig.2.DB79_length_448_mean_cov_17.703125.docx]

**DB79_length_448_mean_cov_17.703125**

```
AGTCTTCCCCAAGCCTTTTCTACTCCTCCCATCTGGAGTTTATCATTCCCTGCTCTGGGTCTTAATCATTTGTGTAGTCTCTTCTTATC
 >chr10:98175132-98175532 + E=1e-223 p=0e+00
ATACTGTATTGTTTTTCTTAAGGAGTGATGGTATTTGTCTTCTTTGCTAAAAGTAAGTTCCTCAAGCAGAGAGACTGGGTGTTTTATCT

CTGTCCTTAGAAACTACAGCACAGCAGACCTCAAAAAATGCTTCTTGAATTAAATATGCAATCAGGATAAAGCACTGTCTTTTAAAAAC

TTTGCAAAATAGTTATTTACATCCAAAATGGAAGTGAAAATGTACATATGTCACCTAGCTATTAGCTGTGCATGCTCTCTCTCTCTGTC

TCTCTCTGTCTCTCTCTCTCTCTCTCTCTCTCTCTCT|CTCTCTC|TCTCTCTCTCTCTCTCTCTCTCTCCCCCCTCCGGCCCCCT|AC
                                      >chr2:168813206-168813253 + E=1e-07
ACCCCT
```
